# Experimental data on water vapour adsorption on silica gel in fully packed and Z-annulus packed beds

**DOI:** 10.1016/j.dib.2021.106736

**Published:** 2021-01-09

**Authors:** Siegfried K. Yeboah, Jo Darkwa

**Affiliations:** a Department of Architecture and Built Environment, Faculty of Science and Engineering, The University of Nottingham Ningbo China, 199 Taikang East Road, Ningbo 315100, PR China; b Department of Architecture and Built Environment, The University of Nottingham, University Park, Nottingham NG7 2RD, UK

**Keywords:** Adsorption data, Data validation, Design parameters, Heat and mass transfer data, Packed bed system, Porous materials, Silica Gel Properties: Solid Desiccants, BET, Brunauer–Emmett–Teller, DSC, Digital Scanning Calorimeter, Di, Inner Diameter, Do, Outer Diameter, FPB, Fully Packed Bed, LAPB, Large Annulus Packed Bed, MAPB, Medium Annulus Packed Bed, MTZ, Mass Transfer Zone, SAPB, Small Annulus Packed Bed, TC, Thermocouple

## Abstract

Experimental data on water vapour adsorption on silica gel in four packed bed configurations namely fully packed bed (FPB), large annulus packed bed (LAPB), medium annulus packed bed (MAPB) and small annulus packed bed (SAPB) are presented. Raw temperature data from designated mass transfer zones (MTZ) in the packed beds and on their corresponding walls are presented along with data of the inlet and outlet moist air conditions. Pressure transducers installed at the inlet and outlet provided pressure data. The presented data also covers the material properties of the silica gel for adsorption obtained through material testing and analysis in the laboratory.

With detailed experimental methodology and comprehensive material and water vapour adsorption data, this article can help other researchers to validate and verify the performance of their adsorption systems. The material property data presented can also help investigators to use appropriate experimentally determined property values of silica gel in their theoretical studies. Furthermore, this data can serve as a basis of comparison for heat and mass transfer in other experimental adsorption systems.

**Specifications Table**

**Table:** 

Subject	Materials Science
Specific subject area	Heat and Mass Transfer
Type of data	Table
	Image
	Chart
	Graph
	Figure
How data were acquired	Surface properties of the silica gel (Silicon Dioxide/S_i_O_2_) particles were determined using Micromeritics ASAP 2020. Test samples of the silica gel particles were weighed with the Mettler Toledo Scale Balance. Material density of the silica gel was determined using Quantachrome Ultra PYC 1200e gas pycnometer. The specific heat capacity of the silica gel particles was determined using the EXSTAR SII DSC 6220 differential scanning calorimeter. Thermal Conductivity (K), Thermal Resistivity (ρ), Volumetric Specific Heat (C) and Thermal Diffusivity (D) of the silica gel particles were determined using the KD2 Pro-device. The silica gel particles were separated into different particle sizes using meshes and their masses determined using a HENGPING Scale Balance. They were then conditioned for adsorption using BPG-9070A electric drying oven. Inlet and outlet air conditions were determined using Sentry ST732 Hotwire Anemometers and AZ 8829 Temperature and RH% data loggers. Inlet and outlet pressures were obtained from QEALY differential pressure metre connected to the Yokogawa MV2000 Data Logger and Personal Computer. Primary packed bed temperature data were obtained from Omega K type thermocouples connected to Yokogawa MV 2000 data logger and Personal Computer.
Data format	Raw
Parameters for data collection	Data was collected under ambient laboratory conditions. Standard atmospheric pressure was approximately 101,325 Pa. Average ambient laboratory temperature ranged between 21 and 26 °C for the various packed bed tests.
Description of data collection	The primary adsorption data was partly obtained from Omega K type thermocouples inserted in three designated mass transfer zones respectively and partly from the inlet and outlet relative humidity obtained using AZ 8829 Temperature and RH% data loggers. Heat transferred from the walls was determined from Omega K type thermocouples attached to the adjoining walls of the mass transfer zones. The Omega K type thermocouples were connected to the Yokogawa MV 2000 data logger which was connected to the Personal Computer to log the raw temperature data every 10.00 s.
	The AZ 8829 Temperature and RH% data loggers were connected to the Personal Computer via their docking stations for transient inlet and outlet temperature and relative humidity data to be logged. The Sentry ST732 Hotwire Anemometers connected to the Personal Computer was used to collect inlet and outlet air temperature and velocity data. Data from the pressure transducers connected to the inlet and outlet of the packed beds were obtained through the QEALY differential pressure metre connected Yokogawa MV 2000 data logger linked to the Personal Computer as electrical signals in Volts which were then converted to Pascals using a conversion factor.
	Data on the pore and surface properties of the silica gel were obtained from BET measurements while thermal property data were obtained from DSC measurements and the thermal properties analyser. True volume and density of the silica gel were determined from gas pycnometer measurements.
Data source location	Institution: The University of Nottingham Ningbo China
	City/Town/Region: Ningbo, Zhejiang Province.
	Country: PR China
Data accessibility	Repository name: Mendeley Data
	Data identification number: http://dx.doi.org/10.17632/xzss43z4fw.3
	Direct URL to data: http://dx.doi.org/10.17632/xzss43z4fw.3
Related research article	S. K. Yeboah, J. Darkwa. Experimental investigations into the adsorption enhancement in packed beds using Z-Annular flow configuration. International Journal of Thermal Sciences. February 2019. Volume 136. Pages 121–134. https://doi.org/10.1016/j.ijthermalsci.2018.10.027

**Value of the Data**  

• The raw primary experimental data presented here can be valuable in the evaluation of the adsorption performance of silica gel-water systems. The data can be used in the validation of other theoretical and experimental studies. The silica gel property data obtained from the various material property techniques such as the BET measurements and the DSC measurements can serve as reference data for other studies.
• Researchers, experts, and industry practitioners interested in heat and mass transfer in solid desiccant adsorption systems can benefit from such data in the validation of their theoretical models and for comparative studies.
• The parameters and the methodology outlined in this article will be useful for those who want to replicate this study. The adsorption performance data can help with comparative studies, model verification and validation.
• Typical solid desiccant dehumidification systems have their own limitations. One such limitation is the heat of adsorption released that limits the adsorption performance of the system. In this study, various configurations of a typical packed bed system were used in order to evaluate their adsorption performances. The key findings here can inform how we thermally manage typical solid desiccant systems for instance in refrigeration/heat pump applications, air pollution control and other adsorption and gas separation applications.

## 1 Data Description

The data presented here are raw primary experimental adsorption data for a fully packed bed (FPB) [1] and three Heggs’ [2] Z-annulus packed bed configurations described by their annular dimensions as small (SAPB), medium (MAPB) and large (LAPB) corresponding to diametrical ratios (Do/Di) of 2, 2.35 and 3.08, respectively. The packed beds were filled with silica gel (Silicon Dioxide/S_i_O_2_) of particle size between 3.35 and 4.75 mm. Inlet moist air condition for adsorption was generated from a purpose-built mixing box incorporating a convective fan heater and a domestic humidifier.

The raw primary experimental adsorption data are in the Mendeley repository as Excel files [3] captioned FPB Primary Data, LAPB Primary Data, MAPB Primary Data and SAPB Primary Data, respectively. Two experimental test runs of each of these adsorption data is presented with the inscription Run 1 or Run 2 after the caption. Each Excel file contains temperature data from three mass transfer zones (MTZ) designated on the packed beds and their adjoining walls, all obtained using Omega K-type thermocouples. Inlet and outlet air conditions of temperature, velocity and relative humidity obtained using Sentry ST732 Hotwire Anemometers and AZ 8829 Temperature and RH% data loggers and differential pressure obtained using QEALY differential pressure metre connected to the Yokogawa MV2000 Data Logger and a Personal Computer are also presented. Additional graphical plots of the inlet and outlet moist air temperature and relative humidity obtained from the AZ 8829 Temperature and RH% data loggers are also presented in the Mendeley repository[3] for each packed bed configuration and corresponding test run.

In the same Mendeley repository [3], material property data of the silica gel, determined from Surface BET measurements, DSC measurement, and thermal analysis using the KD 2 Pro-device are presented in Excel. Analysis reports on true volume and density measurements obtained from the Quantachrome Ultra PYC 1200e gas pycnometer are also available in PDF in the Mendeley repository [3].

### 1.1 BET measurement results

The silica gel (Silicon Dioxide/SiO_2_) particles were tested to ascertain their adsorption capacity. Table 1 shows the surface area, pore volume and pore size obtained after the analysis with the Micromeritics ASAP 2020. The comprehensive data in Excel along with a summary report of the data can be found in the Mendeley Repository [3] as Excel file captioned “*BET Measurement Data – Silica Gel”* and a .png file captioned *“BET Measurement Summary Report”* respectively.

**Table 1:** BET measurements of the silica gel particles from the micromeritics ASAP 2020.

Parameter	Value	Units
BET Surface Area	600.89	m^2^/g
BET Pore Volume	0.35	cm^2^/g
BET Average Pore Width	23.08	Å

**Fig. 1:**
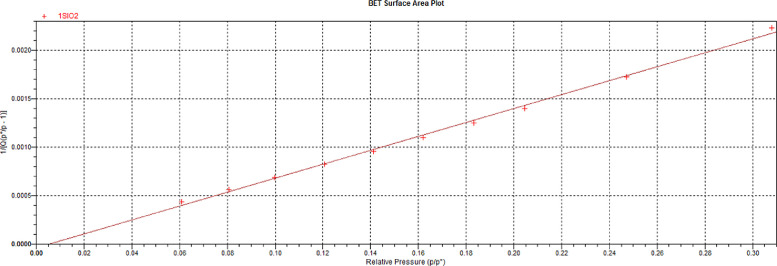
BET Surface Area Plot for the Silica Gel (SiO_2_).

The BET surface area plot in Fig. 1 shows that the adsorption isotherm is linear between relative pressures of 0.01 and 0.32. The related data for this plot can be found in the Mendeley repository [3] in the Excel file captioned *“BET Measurement Data – Silica Gel”*.

**Fig. 2:**
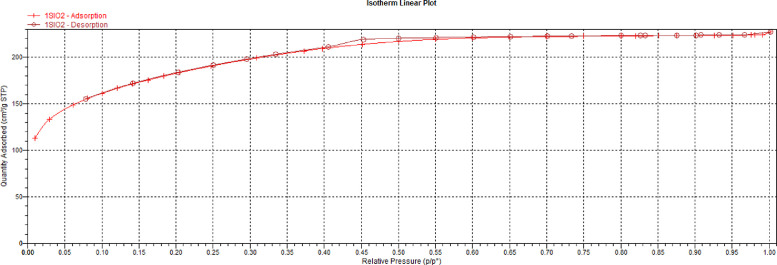
Linear Isotherm Plot for the Silica Gel (SiO_2_).

The linear isotherm plot of the nitrogen adsorptive on the silica gel (SiO_2_) is shown in Fig. 2 with a hysteresis loop between relative pressures of 0.4 and 0.55. The related data can also be found in the Mendeley repository [3] in the Excel file captioned *“BET Measurement Data – Silica Gel”*.

### 1.2 Thermal analysis results

The thermal analysis of the silica gel determined using the KD2 Pro-device generated the following material properties in Table 2. In the Mendeley repository [3] complete data is presented in an Excel file captioned *“Thermal Analysis Data – Silica Gel”.*

**Table 2:** Measured thermal properties of the silica gel using KD2 Pro.

Parameter	Temperature range	Thermal conductivity (K)	Thermal resistivity (ρ)	Volumetric specific heat (C)	Thermal diffusivity (D)	Error margin
U nits	°C	W /(m•K)	°C•cm/W	MJ/(m^3^K)	mm^2^/s	–
Value	26.770–30.049	0.197	506.7	1.703	0.116	0.0007

### 1.3 Pycnometer results

The true volume and density of the silica gel samples were determined using the Quantachrome Ultra PYC 1200e gas pycnometer. Three samples were weighed and analysed using this equipment. Three runs per sample were carried out and the averages of these runs are presented in Table 3. The complete analysis reports for the three runs are presented as PDF files in the Mendeley repository [3] captioned *“Analysis report (1, 2 or 3) on Silica Gel-Quantachrome Ultra PYC 1200e gas pycnometer”.*

**Table 3:** Pycnometer test results.

Sample	Average Volume (cc)	Volume Standard Deviation (cc)	Average Density (g/cc)	Density Standard Deviation(g/cc)	Coefficient of Variation%	Requested Deviation%	Achieved Deviation%
1	34.4029	1.2251	2.2319	0.0858	3.7809	0.0100	3.3605
2	29.8618	2.5778	2.5462	0.2311	8.6325	0.0100	7.7844
3	31.1253	1.3903	2.3202	0.1053	4.4667	0.0100	3.9048

### 1.4 DSC specific heat plot, cp

**Fig. 3:**
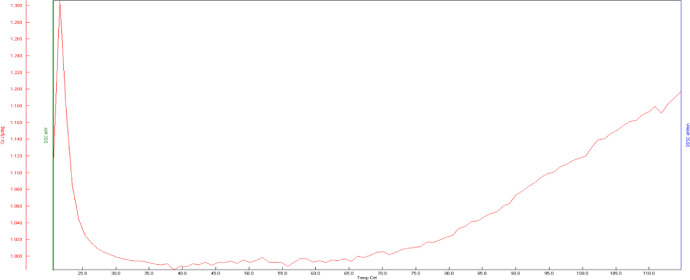
Plot of specific heat capacity against temperature for the silica gel.

The specific heat capacity of the silica gel particles was determined from room temperature to the regeneration temperature of the silica gel. Fig. 3 shows the DSC plot for the cp obtained for the silica gel using the EXSTAR SII DSC 6220. The comprehensive test results and conditions are provided on an Excel Spreadsheet as supplementary material captioned *“Silica Gel DSC Results”* in the Mendeley repository [3]. This spreadsheet has three coloured tabs one for the silica gel analysis condition, the DSC Chart, and the specific heat values of the silica gel at the various temperatures during testing.

### 1.5 Empty and real bed velocities

The empty bed and real bed velocities were determined by allowing the inlet air to go through the empty packed bed and the random densely packed bed of silica gel particles for all packed bed configurations. The inlet and average outlet velocity obtained for the FPB when empty and when randomly filled with silica gel particles can be found in Yeboah and Darkwa [1]. For the annulus packed beds, Table 4 shows the average inlet and outlet velocities when randomly filled with the silica gel particles.

**Table 4:** Annulus packed bed testing results.

Packed Bed	Average Inlet Velocity, m/s	Average Outlet Velocity, m/s	Comments
LAPB	0.47	0.45	• Random and densely packed silica gel particles.
MAPB	0.44	0.52	• Average inlet and outlet velocities.
SAPB	0.30	0.45	

### 1.6 Inlet conditions

The inlet moist air conditions for the packed beds were generated using the mixing box designed and fabricated purposely for these experimental investigations. The Sentry ST 732 Hotwire Anemometer obtained the inlet air temperature and velocity whilst the AZ 8829 sensor and data logger collected the relative humidity and inlet air temperature data. Plots for this can be found in Yeboah and Darkwa [1] and the raw primary data in the Mendeley Dataset [3].

## 2 Experimental Design, Materials and Methods

### 2.1 Silica gel testing

The Silicon Dioxide (SiO_2_)/silica gel particles obtained from a local manufacturer for this study came with only a small amount of data mainly on the range of the particle sizes and a limited thermophysical property. To ascertain its adsorption capacity and suitability for the investigation, the following material testing and verification of properties was carried out:

• Surface BET Test
• Density Measurement
• Specific Heat Capacity Measurement
• Material Thermal Analysis

#### 2.1.1 The surface bet test

The surface properties of the particles were determined using the Brunauer-Emmett-Teller (BET) method, most widely used standard procedure for the determination of the surface area of finely divided and porous materials [4]. This was achieved using the Micromeritics ASAP 2020, a Surface Area and Porosity Analyser. Nitrogen at 77 K was used as it is considered a standard adsorptive for surface area and pore size analysis [5]. First, the test tube holder was weighed separately and then together with the empty test tube using the Mettler Toledo Scale Balance. A small sample was then put in the test tube using a spatula and also weighed. The various weights were recorded, and the sample weight determined as shown in Table 5. The sample was then ready for degassing using the Micromeritics ASAP 2020. The first stage of the degassing process was done at 90 °C and then at 350 °C for about 5–6 h.

**Table 5:** Sample weighing data.

Item	Weight	Units
Test Tube	37.2206	g
Test tube + Sample Before Degassing	37.8034	g
Test tube + Sample After Degassing	37.7521	g
Sample Weight After Degassing	0.5315	g

After degassing, the sample was weighed again on the balance for surface area and porosity analysis using Micromeritics ASAP 2020. The test tube with the degassed sample was then connected to the Micromeritics ASAP 2020. The analysis pots for the Micromeritics ASAP 2020 were filled with liquid nitrogen and placed on their holders. A sample analysis file was then created on the computer and analysis was commenced. The analysis was carried out at a bath temperature of −198.8 °C. The BET Measurement Data – Silica Gel Excel file in Mendeley repository [3] has further details on the measurement conditions.

#### 2.1.2 Density measurement

The Quantachrome Ultra PYC 1200e gas pycnometer was used to measure the true volume of the silica gel particles by employing Archimedes’ principle of fluid displacement and gas expansion using Boyle's Law [6]. The analysis was carried out using Helium gas at a pressure of about 120 kPa. Three test samples were used. Before the analysis, the samples were weighed on a balance and the weights entered in the pycnometer. The analysis temperature was 34.3, 34.0 and 33.7 °C respectively for the samples. For each sample, a maximum of three test runs were carried out. In the Analysis Report (1, 2 or 3) on Silica Gel-Quantachrome Ultra PYC 1200e gas pycnometer PDF files in the Mendeley repository [3] the sample and analysis parameters are comprehensively provided along with the results of the measurements*.*

#### 2.1.3 Specific heat capacity measurement

The differential scanning calorimeter was used to determine the specific heat capacity of the silica gel particles. The procedure involved a small sample weighed and heated in the EXSTAR SII DSC 6220, to dry the sample before the measurement and analysis. An empty vial and a vial containing a sample of sapphire were used as measurement references. The Excel file captioned “*Silica Gel DSC Results”* in the Mendeley repository [3] contains further details on the sample parameters as well as the test conditions. These parameters could both be found via the silica-gel-analysis tab (colour coded green) and the DSC Chart tab (colour coded blue) in the Mendeley repository [3].

#### 2.1.4 Material thermal analysis

Other thermal properties of the silica gel particles were determined using KD2 Pro, a battery-operated, menu-driven device that measures thermal conductivity and resistivity, volumetric specific heat capacity and thermal diffusivity. To measure the properties of interest, a dual needle (sensor) of dimensions 1.3 mm diameter x 30 mm long, 6 mm spacing was inserted into a beaker full of the silica gel particles. The measurement was carried out when the needle temperature was in equilibrium with the surrounding air temperature. The measurement took approximately 2 min at 60 temperature points between 26.770 and 30.049 °C. Prior to measurement the silica gel particles was dried in the BPG-9070A drying oven for about 3 h at a temperature of about 115 °C and left to cool in the oven overnight. This was to ensure that any physisorbed water vapour was removed before measurement commenced. The measuring range for the KD2 Pro-is 0.02 to 2.00 W/(m • K) for thermal conductivity, 50 to 500 °C • cm/W for thermal resistivity, 0.1 to 1 mm^2^/s for thermal diffusivity and 0.5–4 mJ/(m^3^K) for volumetric specific heat with *a* ± 10% accuracy for all of the properties it measures. The testing process involved a beaker filled to the brim with the silica gel particles and then the dual sensor pins inserted vertically in them.

### 2.2 Silica gel preparation for adsorption

#### 2.2.1 Silica gel sieving

The silica gel particles were of varied sizes hence there was the need for them to be sieved into specific size categories. The sieving process was undertaken manually in a fuming cupboard to prevent dust particles from being inhaled and settling on surfaces in the laboratory. The mesh sizes used were 4.75 mm, 3.35 mm, 2.36 mm and 1.70 mm. For the study, the particle size used was between 3.35 mm-4.75 mm.

#### 2.2.2 Silica gel drying

The silica gel particles were conditioned for adsorption by drying in the BPG-9070A electric oven. The particles were initially weighed and then put in trays, spread out in thin layers for the drying process. The drying oven was set to a maximum temperature of 140 °C. The silica gel particles were dried for a period between 4 and 5 h and allowed to cool before being packed in the vessels. To determine how much moisture had been removed by the drying process, the silica gel particles were weighed after being removed from the oven and the difference in weights recorded.

### 2.3 The packed bed system

#### 2.3.1 Physical model

**Fig. 4:**
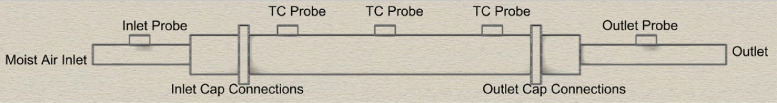
Schematic of packed bed with annotations.

The packed bed is a simple cylindrical vessel with two caps at both ends for the inlet and outlet sections as shown in Fig. 4. Inside the bed is a copper mesh designed to hold the silica gel particles in place.

The annulus section was designed as cylindrical mesh pipes for central placement in the packed bed to allow the silica gel particles to be packed around them to ensure radial flow of moist air within the bed as shown in Yeboah and Darkwa [1].

#### 2.3.2 The fabricated packed bed and components

The packed bed was made of copper of thickness 1 mm with the inlet and outlets designed as caps to fit on the main cylindrical vessel. The cylindrical vessel was fitted with two meshes close to the inlet and outlet to hold the silica gel particles in place in order to maintain the 30 cm packing length. Each mesh was held in place by four (4) small screws drilled through the walls of the bed. Thermocouples were inserted into the inlet probes on the bed vessels made from three small holes equally spaced apart in a straight line on the vessel. Two of these holes were 5 cm from the inlet and outlet respectively and one placed exactly on the 15 cm mark on the vessel.

**Fig. 5:**
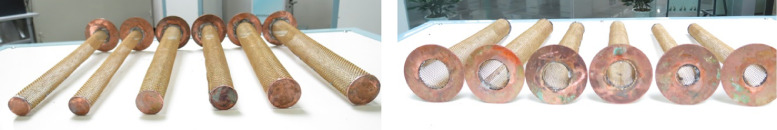
Different dimensions of the copper cylindrical mesh pipes fabricated for the experiment.

The cylindrical mesh pipes as presented in Yeboah and Darkwa [1] were made from copper mesh of size 0.08 m as shown in Fig. 5. They were capped at one end with a copper plate to ensure the distribution of the moist air is radial within the annulus section, a configuration consistent with the Heggs et al. [2] “Z” arrangement.

#### 2.3.3 Empty and real bed velocities

The packed beds were tested empty and then with packed silica gel particles in order to determine the empty and real bed velocities respectively.

#### 2.3.4 Determination of packing porosity

The mean porosity for the packing was determined using two volumetric cylinders and a scale balance. An amount of dry silica gel particles was placed in one of the volumetric cylinders and the volume and mass determined. A known quantity of water was then added to the dry silica gel particles in the volumetric cylinder and the new mass and volume determined. The packing density and the particle density were then used to determine the packing porosity. Data relating to the packing porosity determination can be found in Yeboah and Darkwa [1].

### 2.4 Moist air mixing box design and fabrication

**Fig. 6:**
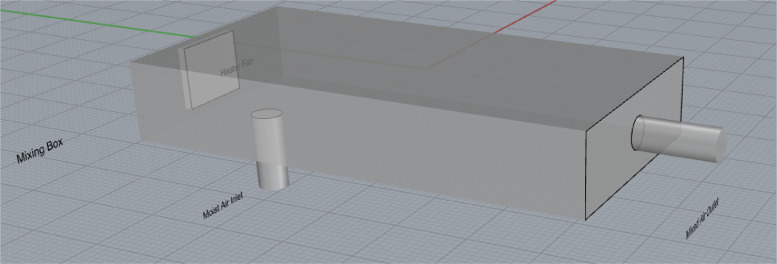
Ghosted image of the mixing box designed in rhinoceros 5.

The inlet air condition for the adsorbing packed bed was generated using a moist air mixing box comprising of an inlet for a humidifier and a convective fan heater. The physical model is shown in Fig. 6 and dimensions in Table 6.

**Table 6:** Mixing box dimensions.

Parameter	Value	Units
Moist Air Inlet Diameter	40	mm
Moist Air Inlet Length	100	mm
Mixing Box Outlet Diameter	80	mm
Mixing Box Outlet Length	100	mm
Fan Heater Length	124	mm
Fan Heater Width	76	mm
Box Length	600	mm
Box Width	300	mm
Box Height	100	mm

The mixing box was fabricated out of uPvc material integrated with a 300 W convective fan at the back. It was then connected to a 5 L domestic humidifier via a flexible pipe through the internal opening at iits bottom. The mixing box was tested to determine any variations in flow rate and relative humidity of the air mixture using the Sentry ST732 Hotwire Anemometer. The humidifier inlet dimension was further reduced from its original design to about 0.5 cm so the amount of moisture can be properly controlled.

### 2.5 The adsorption experiment

The experiments began with the preparation of the silica gel particles. This involved sieving to get the particles in the appropriate size range, determination of its mass before drying, drying at a maximum temperature of 140 °C in the BPG-9070A electric oven for 4–5 h and allowing the silica gel particles to cool. The cooled silica gel was then weighed again. The mass of the silica gel was determined using the HENGPING electronic scale balance.

**Fig. 7:**
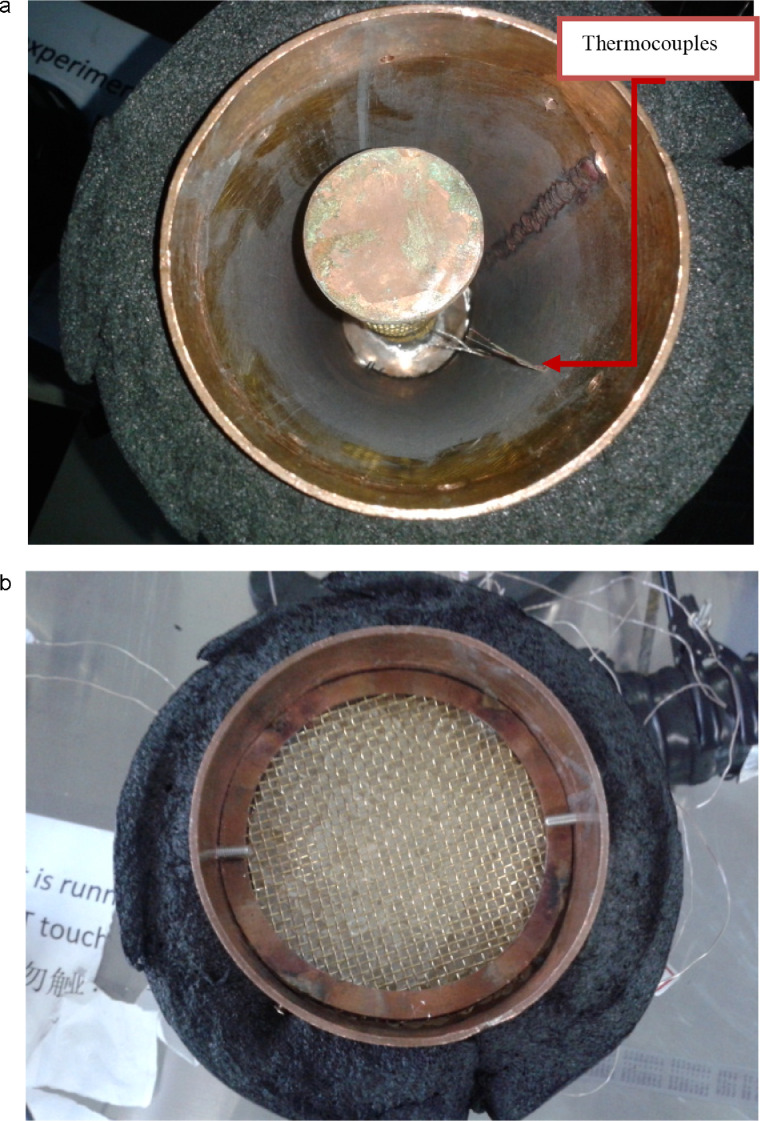
(a) Packed bed with annulus insert and thermocouple (TC) Probes. (b) insulated vessel with silica gel packing.

The annulus inserts were placed centrally in the bed (See Fig. 7a) and the silica gel particles randomly packed around them (See Fig. 7b). For each experimental run, the packed bed was insulated with a 20 mm thick nitrile rubber thermal insulation material. At the interior and the walls of the packed bed temperature measurement were taken using Omega K-type thermocouples. The thermocouples were inserted at three points in the packed bed creating three mass transfer zones of interest of equal distances apart, referred to as MTZ 1, MTZ 2 and MTZ 3 respectively. These thermocouples were connected to a Yokogawa MV2000 Data Logger (See Fig. 7a) for temperature measurements typically within the packed beds and on the cooresponding walls.

For the inlet and outlet relative humidity and temperature measurements, AZ 8829 Temperature and RH% data loggers were used. These data loggers were inserted in flexible pipes connecting the inlet and outlet of the adsorbing bed. Inlet and outlet velocities and also air temperature were measured using Sentry ST 732 Hotwire Anemometers. Pressure probes were inserted in the inlet and outlet of the adsorber for pressure measurement using the QEALY differential pressure metre. The QEALY differential pressure metre was then connected to the Yokogawa MV2000 Data Logger connected to the Personal Computer to measure the inlet and outlet pressures through electrical pulses in Voltage. The results obtained were then converted into Pascal's using a conversion factor.

The inlet of the packed bed was connected to the mixing box where moist air was allowed to flow through the inlet into the respective bed configurations for adsorption. Sensors were placed at the inlet, outlet to measure the velocity, pressure, relative humidity and temperature. The data loggers were set to obtain data at 10.00 s interval to enable very small changes in bed temperature and other measuring parameters during the adsorption process to be observed.

## Ethics Statement

Not Applicable

## CRediT Author Statement

**Siegfried K. Yeboah:** Conceptualization; Data curation; Formal analysis; Investigation; Methodology; Project administration; Resources; Roles/Writing - original draft; Validation; Visualization. **Jo Darkwa:** Writing - Review & Editing and Supervision.

## Declaration of Competing Interest

The authors declare that they have no known competing financial interests or personal relationships which have, or could be perceived to have, influenced the work reported in this article.

## Appendix Supplementary materials

Image, application 1
